# Association of AGTR1 (rs5186), VDR (rs2228570) genes polymorphism with blood pressure elevation in patients with essential arterial hypertension

**DOI:** 10.25122/jml-2021-0018

**Published:** 2021

**Authors:** Marianna Mykolaivna Semianiv, Larysa Petrivna Sydorchuk, Valentyna Stepanivna Dzhuryak, Oleg Vasylovich Gerush, Oleg Vasylovich Gerush, Alina Oleksandrivna Palamar, Natalia Yaroslavivna Muzyka, Oksana Mykolaivna Korovenkova, Olesia Mykhaylivna Blazhiievska, Valeriia Valeriivna Sydor, Andrii Ruslanovich Sydorchuk, Igor Oleksandrovich Semianiv, Ruslan Igorovich Sydorchuk

**Affiliations:** 1. Family Medicine Department, Bukovinian State Medical University, Chernivtsi, Ukraine; 2. Pharmacy Department, Bukovinian State Medical University, Chernivtsi, Ukraine; 3. Emergency and Trauma Surgery Department, St. Anna Hospital, Herne, Germany; 4. Phthisiology and Pulmonology Department, Bukovinian State Medical University, Chernivtsi, Ukraine; 5. General Surgery Department, Bukovinian State Medical University, Chernivtsi, Ukraine

**Keywords:** essential arterial hypertension, gene polymorphism, AGTR1 (rs5186), VDR (rs2228570), EAH – Essential arterial hypertension, BP – Blood pressure, AGTR1 – Angiotensin II receptor type 1, VDR – Vitamin D receptor, CVD – Cardiovascular diseases, RAAS – Renin-angiotensin-aldosterone system, SBP – Systolic blood pressure, DBP – Diastolic blood pressure, BMI – Body mass index, WC – Waist circumference, WHR – Waist-to-hip ratio

## Abstract

Essential arterial hypertension (EAH) is a polygenic disease due to environmental, genetic, and epigenomic factors. The study aimed to establish the association of single nucleotide polymorphism (SNP) of AGTR1 (rs5186) and VDR (rs2228570) genes with the blood pressure (BP) elevation in EAH patients. 100 EAH subjects with hypertensive-mediated organ damaging (2^nd^ stage), moderate, high, or very high cardiovascular risk were recruited into the case-control study. There were 70.83% females and 29.17% males, mean age 57.86±7.81 y.o. The control group included 60 healthy individuals of relevant age and gender distribution. Estimation of AGTR1 (rs5186) and VDR (rs2228570) gene polymorphism was performed by Real-Time Polymerase Chain Reaction. In EAH patients, the AGTR1 gene (rs5186) mutation occurs with a frequency of 2.78% in the absence of such among healthy individuals. The VDR (rs2228570) gene mutation occurs with a frequency of 23% cases. The C-allele carriers’ (AGTR1 gene) numbers with 2^nd^ and 3^rd^ BP values degree dominate over AA-genotype patients by 25.32% (_χ_2=4.52; p=0.033). VDR gene (rs2228570) polymorphic variants do not link to BP elevation values. Thus, the C-allele of the AGTR1 gene (rs5186) is associated with BP elevation in hypertensive patients. BP values do not depend on VDR gene (rs2228570) polymorphic variants.

## Introduction

Essential arterial hypertension (EAH) affects about 25–43% of the population worldwide. Medical experts estimate that EAH is the most common disease globally: from 1 billion people in 2000 to 1,6 billion in 2025 [1–3]. Also, EAH is one of the most important cardiovascular and metabolic risk factors. As a polygenic disease, arterial hypertension occurs due to the environmental and genetic predictors interactions in linkage with epigenomic structures. Most of the genetic variations associated with EAH that were collected in Genome-Wide Association Studies (GWASs) [4–5], where SNPs which play the role of possible biomarkers for hypertension screening [5–8]. The renin-angiotensin-aldosterone system (RAAS) is a complex multilevel mechanism regulating vascular tone, metabolic homeostasis, water, and electrolyte balance. Therefore, the study of RRAS components, particularly the vasoconstrictor’s genetic marker like angiotensin II receptor type 1 (AGTR1) and metabolic regulator – vitamin D receptor (VDR), is essential for the future prevention of possible EAH progression and complications. Several studies have shown the association of AGTR1 (1166A>C, rs5186) gene polymorphism with hypertension, vasoconstriction, and sodium retention in the body [9, 10]. Recent studies have demonstrated that vitamin D deficiency is associated with skeletal diseases and other chronic pathologies, including cardiovascular disease (CVD) and EAH. Vitamin D may downregulate the RAAS system activity and might be a possible key factor of blood pressure (BP) control [11]. Some studies have found that low vitamin D levels may be associated with EAH, high BP, gestational hypertension, and preeclampsia [12–16]. Wang L. *et al.* [17] found that VDR polymorphisms BsmI and FokI are linked to hypertension in the US population. Even though genetic predictors of RAAS activity, including EAH, have been studied widely throughout the last decade [1, 10, 18–22], a number of them remain unexplored, especially in the Ukrainian population.

The study aimed to establish the association of SNP polymorphism of AGTR1 (rs5186) and VDR (rs2228570) genes with the BP elevation values in EAH patients.

## Material and Methods

**Figure 1.**
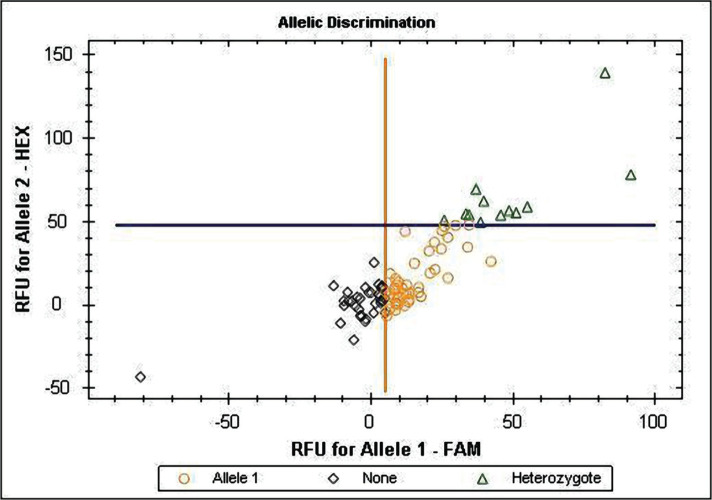
Alleles discrimination of the AGTR1 gene polymorphism (1166A>C). ο Allele 1 – homozygous carriers of the A-allele; • Allele 2 – homozygous carriers of the C-allele; Δ Heterozygote – carriers of AC genotype; ◊ None - indefinite.

**Figure 2.**
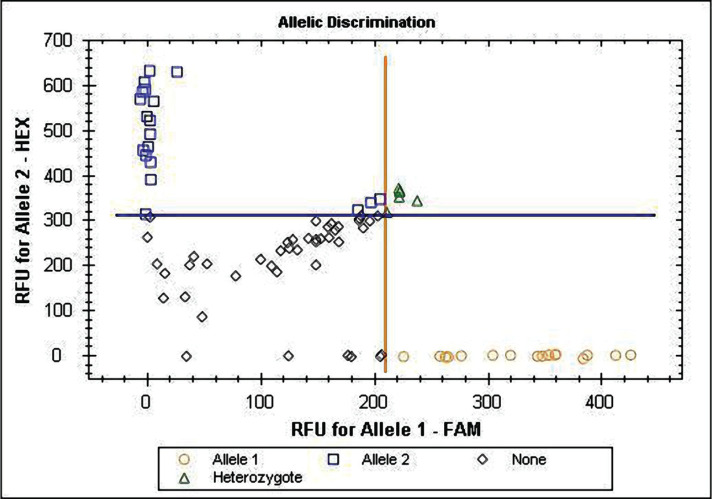
Alleles discrimination of the VDR gene (rs2228570) polymorphism. ο Allele 1 – homozygous carriers of the A-allele; • Allele 2 – Allele 2 - homozygous carriers of the G-allele; Δ Heterozygote – Heterozygote - carriers of AG-genotype; ◊ None - indefinite.

Hypertension was defined as office systolic blood pressure values ≥140 mmHg and/or diastolic BP (SBP, DBP) values ≥90 mmHg at least for three measurements during a month, according to national and European Societies of Hypertension and Cardiology (ESH/ESC, 2016, 2018) recommendations requirement. All enrolled patients were observed by family physicians and cardiologists and underwent a complex of basic examinations: general clinical analyses of CBC, cholesterol panel, Body Mass Index (BMI, kg/m^2^) for evaluation of overweight and abdominal obesity, Waist Circumference (WC), Waist-to-Hip ratio (WHR), ECG and EchoCG. After screening for inclusion and exclusion criteria, 100 patients were selected: 70.84% women, 29.16% men, with average age 57.86±7.81 years old. Inclusion criteria: the study included patients with EAH stage II, 1–3 grades of BP elevation, moderate, high, and very high cardiovascular risk (according to ESC recommendation Guidelines 2018); aged 40–70 years. Exclusion criteria were described in our former publications [19–21, 23]. We excluded patients with EAH stage III, with chronic heart failure of the functional class higher than II (NYHA III-IV); secondary arterial hypertension; type I diabetes mellitus, sub- and decompensated type 2 diabetes mellitus (with damage of the target organs); malignant or uncontrolled EAH; sub- and decompensated liver diseases (level of aspartate aminotransferase, alanine aminotransferase three times higher than the normal); bronchial asthma, chronic obstructive pulmonary disease stage III-IV; exacerbation of infectious diseases; mental disorders; oncological diseases; taking oral corticosteroids or contraceptives; pregnancy, or lactation. The control group included 60 healthy individuals: 62.5% women and 37.5% men, with an average age of 57.86±7.81 years old. Subjects of both groups did not differ by gender and age (p>0.05). The genotyping of the analyzed gene polymorphisms was performed by Real-Time Polymerase Chain Reaction (RT-PCR) using the RT-PCR system CFX96 Touch™ (BioRad, USA). Vacutainer tubes containing ethylenediaminetetraacetic acid were used to collect the venous blood of the examined. Extraction of DNA from the nuclei of lymphocytes of patients was performed according to the Thermo Scientific GeneJET Genomic DNA Purification Kit (Thermo Fisher Scientific, USA), as described in our former publications [19–21, 23]. Alleles discrimination of AGTR1 (rs5186) and VDR (rs2228570) gene polymorphisms were provided by licensed computer Software Bio-Rad RealTime (Microsoft, USA). The genotype detection results of AGTR1 (rs5186) and VDR (rs2228570) genes are given in Figures 1 and 2. AGTR1 gene analysis was provided for 72 EAH patients and 48 healthy subjects, VDR gene – for 100 EAH patients and 60 healthy people.

Statistical analysis was performed using StatSoft Statistica v. 7.0 (USA) software. Pearson’s criterion (_χ_2) was used for the genotypes distribution comparison. Analysis of qualitative data (categorical variables), risk of pathology development was assessed by a binary logistic regression model using relative risk (RelR). Risk ratio (RR) was estimated by odds ratio (OR) with 95% confidence interval [95% CI] using a chi-square test (_χ_2) (df=1). Quantitative data was calculated using a Student’s t-test (two-tail distribution and equal variances between the two samples) based on the triplicate values for each gene genotype. The Wilcoxon-Mann-Whitney U-test was applied in case of uneven data distribution (according to W-Shapiro-Wilk or Kolmogorov-Smirnov test results). Differences were regarded as significant at p values <0.05.

## Results

Genotypes and allele distribution of AGTR1 (rs5186) and VDR (rs2228570) genes polymorphism in patients and in the control group did not significantly differ (Table 1). The wild A-allele of AGTR1 (rs5186) gene dominated over the C-allele: in EAH patients 3.8 times [OR=14.44; 95%OR: 8.18-25.50; p<0.001], in the control group 5.9 times [OR=34.31; 95%OR: 15.39-76.47; p<0.001]. The occurrence of minor A-allele of VDR (rs2228570) gene was by 4% less frequent than G-allele in the study group (p>0.05) and by 5.33% less frequent than in the control group (p>0.05).

**Table 1.** Genotypes and alleles distribution of AGTR1 (rs5186) and VDR (rs2228570) genes polymorphism in observed subjects.

Genotypes, alleles, n (%)		Study group, (%)	Control group, (%)	OR [95% CI]	_χ_2 p
**AGTR1 gene (rs5186)**					
**AGTR1 (1166A>C), n (%)**	**A**	44 (61.11)	34 (70.83)	0.65 [0.30-1.41]	_χ_2=1.20 p>0.05
	**AC**	26 (36.11)	14 (29.17)	1.37 [0.62-3.01]	_χ_2<1.0 p>0.05
	**CC**	2 (2.78)	0	-	-
**AGTR1 (1166A>C), n (%)**	**A-allele**	114 (79.17)	82 (85.42)	0.65 [0.32-1.30]	_χ_2=1.50 p>0.05
	**C-allele**	30 (20.83)	14 (14.58)	1.54 [0.77-3.09]	
**VDR gene (rs2228570)**					
**VDR (A/G), n (%)**	**GG**	27 (27.0)	14 (23.33)	1.21 [0.58-2.56]	_χ_2<1.0 p>0.05
	**AG**	50 (50.0)	28 (46.67)	1.14 [0.60-2.17]	_χ_2<1.0 p>0.05
	**AA**	23 (23.0)	18 (30.0)	0.70 [0.33-1.43]	_χ_2<1.0 p>0.05
**VDR (A/G), n (%)**	**G-allele**	104 (52.0)	56 (46.67)	1.24 [0.77-1.95]	_χ_2<1.0 p>0.05
	**A-allele**	96 (48.0)	64 (53.33)	0.81 [0.51-1.27]	

OR – odds ratio; CI – Confidence Interval; p – significance of differences.

**Figure 3.**
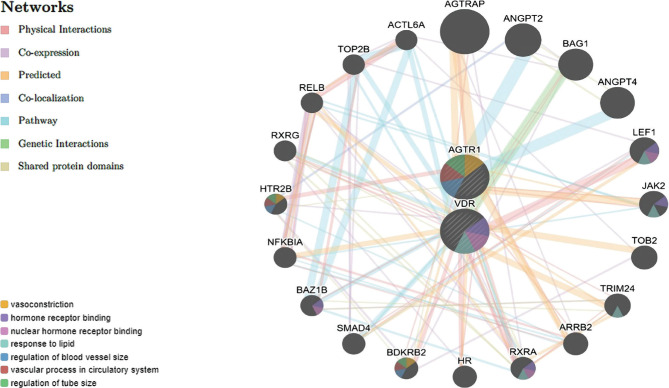
The network of AGTR1 and VDR gene-gene interactions.

The anthropometric and hemodynamic parameters depending on polymorphic variants of the AGTR1 (rs5186) gene are presented in Table 2. We found higher SBP, DBP values in C-allele patients (AGTR1, rs5186) than in AA-genotype carriers (p<0.05). No significant difference in anthropometric parameters depending on the AGTR1 (rs5186) gene genotypes was established. At the same time, the value of SBP and DBP, BMI in women, WC, and WHR in patients dominated the control group for the corresponding genotype by 10.75–38.09% (p<0.05). No reliable differences were obtained in SBP, DBP, BMI, WHR values depending on VDR (rs2228570) gene polymorphic variants (Table 3). Almost all the parameters mentioned above significantly exceeded those in the control group. In patients and healthy GG-genotype carriers the WC value was higher than in A-allele patients by 6.04% (pAA=0.039) and 6.58% (pAG=0.014), in healthy individuals – by 13.79% (pAA=0.025), respectively. The WHR in healthy GG-genotype carriers was higher than in AG-genotype carriers by 10.84% (pAG=0.007), in patients – by 3.33% (pAG>0.05). One-way ANOVA analysis did not confirm the association of the VDR gene (rs2228570) with the anthropometric and hemodynamic parameters. We built a network of AGTR1 (1166A>C) and VDR (A/G) gene interactions using GeneMania, as well as the functional relationships in the constellation of physical interaction, co-expression, co-localization, genetic interaction, shared protein domains, and prediction of such possible interactions (Figure 3). Hypotheses about the functional interaction of genes through vasoconstriction, hormone receptor binding, nuclear hormone receptor binding, response to lipids, regulation of blood vessel size (vascular wall thickness), vascular processes in the circulatory system, regulation of the vascular tube size etc were formed after taking into account the priority of links. The strongest functional links in at least 3–4 functional areas of interaction were observed with the genes HTR2B, BDKRB2, RXRA, JAK2, LEF1 [24–28].

**Table 2.** The anthropometric and hemodynamic parameters depending on AGTR1 (rs5186) gene polymorphic variants, M±m.

**Values**		**AGTR1 gene genotypes in the control group**		**AGTR1 gene genotypes in the study group**	
				**AA-**	**AC+CC-**
**SBP, mm Hg**		**AA-**	117.11±1.14	150.83±2.12 p<0.001	159.80±2.77 p<0.001 p_AA_<0.05
		**AC+CC-**	115.72±2.02		
**DBP, mm Hg**		**AA-**	77.10±1.14	92.60±1.07 p<0.001	97.0±1.85 p<0.001 p_AA_<0.05
		**AC+CC-**	74.30±2.97		
**Body mass index, kg/M^2^**	**M**	**AA-**	27.19±1.49	30.09±1.44	33.09±2.27
		**AC+CC-**	29.85±3.36		
	**F**	**AA-**	25.08±1.24	31.79±0.99 p<0.001	32.50±1.28 p<0.05
		**AC+CC-**	26.0±3.42		
**Waist Circumference, CM**		**AA-**	92.37±2.46	102.30±1.97 p=0.003	103.48±2.37 p=0.008
		**AC+CC-**	86.09±2.58		
**Waist-to-Hip Ratio**		**AA-**	0.87±0.02	0.92±0.02 p<0.05	0.93±0.02 p<0.05
		**AC+CC-**	0.82±0.05		

SBP – systolic blood pressure; DBP – diastolic blood pressure; M – males; F – females; p – significance of differences with control group; pAA – significance of differences with AA-genotype carriers.

**Table 3.** The anthropometric and hemodynamic parameters depending on VDR (rs2228570) gene polymorphic variants, M±m.

Values		**VDR gene genotypes** **in the control group**		**VDR gene genotypes in the control group**		
				**AA**	**AG**	**GG**
**SBP, mm Hg**		**AA**	116.67±1.57	157.0±4.36 p<0.001	152.60±2.32 p<0.001	150.20±2.81 p<0.001
		**AG**	115.0±1.33			
		**GG**	118.57±2.14			
**DBP, mm Hg**		**AA**	76.67±1.0	95.35±2.15 p<0.001	93.90±1.53 p<0.001	94.0±1.34 p<0.001
		**AG**	74.29±1.52			
		**GG**	78.57±2.14			
**Body mass index, kg/M^2^**	M	AA	27.20±2.69	30.02±2.21	29.40±1.43	35.40±2.71 p<0.05
		**AG**	28.20±1.37			
		**GG**	27.10±2.69			
	**F**	**AA**	28.38±0.63	32.10±0.94 p=0.013	31.27±1.33 p=0.011	32.04±1.48 p=0.042
		**AG**	26.65±2.36			
		**GG**	28.88±0.77			
**Waist Circumference, CM**		**AA**	87.0±1.75	99.87±2.05 p<0.001	99.36±2.13 p<0.001	105.90±2.45 p=0.012 p_AA_=0.039 p_AG_=0.014
		**AG**	86.86±2.77			
		**GG**	99.0±1.65 p_AA_=0.025			
**Waist-to-Hip Ratio**		**AA**	0.87±0.03	0.91±0.02 p<0.05	0.90±0.02 p=0.008	0.93±0.01 p_AG_=0.052
		**AG**	0.83±0.02			
		**GG**	0.92±0.01 p_AG_=0.007			

SBP – systolic blood pressure; DBP – diastolic blood pressure; M – males; F – females; p – significance of differences with control group; p_AA_, p_AG_ – significance of differences with AA-genotype or AG-genotype carriers.

The number of C-allele carriers of AGTR1 gene (1166A>C) with the 2^nd^ and 3^rd^ degrees value of BP dominates over AA-genotype patients by 25.32% (_χ_2=4.52; p=0.033) (Table 4). However, AA-genotype patients with high normal BP and the 1^st^ BP grade prevails over the C-allele carriers but insignificantly. The number of patients with the 2^nd^ and 3^rd^ BP elevation degrees does not depend on VDR gene (rs2228570) polymorphic variants (Table 5).

**Table 4.** Blood Pressure values depending on polymorphic variants of the AGTR1 (rs5186) gene.

Blood pressure values, mm Hg		**AGTR1 gene genotypes, n=72 (%)**		**OR** **[95% CI]**	_χ_2 p
		**AA-genotype**	**AC-, CC-genotypes**		
**SBP/DBP, n (%)**	130–139/80–89, n=10	8 (18.18)	2 (7.14)	0.35 [0.07-1.37]	_χ_2<1.0 p>0.05
	1^st^ degree of hypertension, 140–159/90–99, n=32	22 (50.0)	10 (35.71)	0.56 [0.21-1.47]	_χ_2=1.41 p>0.05
	2^nd^, 3^rd^ degrees of hypertension, ≥160/≥100, n=30	14 (31.82)	16 (57.14)	2.86 [1.07-7.62]	_χ_2=4.52 p=0.033

SBP/DBP – systolic/diastolic blood pressure; OR – odds ratio; 95%CI – Confidence Intervals; p – significance of differences.

**Table 5.** Blood Pressure values depending on polymorphic variants of the VDR (rs2228570) gene.

**Blood pressure values, mm Hg**		**VDR gene genotypes, n=100 (%)**			**_χ_2 p**
		**AA-genotype**	**AG-genotype**	**GG-genotype**	
**SBP/DBP, n (%)**	130–139/80–89, n=10	2 (8.70)	5 (10.0)	3 (11.11)	_χ_2<1.0 p>0.05
	1^st^ degree of hypertension, 140–159/90–99, n=52	10 (43.48)	28 (56.0)	14 (51.85)	_χ_2<1.0 p>0.05
	2^nd^, 3^rd^ degrees of hypertension, ≥160/≥100, n=38	11 (47.83)	17 (34.0)	10 (37.04)	_χ_2=1.29 p>0.05

SBP/DBP – systolic/diastolic blood pressure; OR – odds ratio; 95%CI – Confidence Intervals; p – significance of differences.

## Discussion

Mutations in the genes encoding RAAS are being actively studied worldwide, but their role in the pathogenesis of EAH is not entirely elucidated [1, 4, 5, 8, 9, 11]. Numerous studies have shown the association of AGTR1 gene 1166A>C (rs5186) polymorphism with arterial hypertension, vasoconstriction, and sodium retention in the body [9, 10]. We have found a linkage between the C-allele of AGTR1 gene (rs5186) and EAH severity, but much less association between the minor A-allele of VDR gene (rs2228570) and severe EAH course. In some research, no association of the VDR gene with vitamin D levels has been established yet. On the other hand, they have found correlations of the VDR gene with plasma renin concentration [13]. Swapna N. *et al.* [29] suggested that VDR FokI polymorphism is an EAH risk factor in the population of India. In our former studies, we proved the association of BP elevation with some genetic polymorphisms [10, 19–21, 23]. Lopez-Mayorga *et al.* did not confirm the linkage of VDR gene three polymorphisms with cardiometabolic risk. However, the study was performed on children [30]. Nevertheless, the conclusion about the association of AGTR1 (rs5186) and VDR (rs2228570) with EAH is questionable and differs in populations due to various designs and patterns of research [31]. However, further studies might support this issue. A possible limitation of the study is the number of enrolled subjects.

## Conclusion

The C-allele of the AGTR1 gene (rs5186) is associated with BP elevation in hypertensive patients. BP values do not depend on VDR gene (rs2228570) polymorphic variants. BMI, WC, and WHR do not depend on AGTR1 (rs5186) gene polymorphism. Although, the GG-genotype of the VDR gene (rs2228570) is associated with higher WC in both hypertensive and healthy subjects

## Acknowledgments

### Conflict of Interest

The authors declare that there is no conflict of interest.

### Ethics approval

The work was performed according to the requirements for research of the Statute of the Ukrainian Association for Bioethics and the GCP norms (1992), requirements and norms of ICH GLP (2002), ethical standards in the Helsinki Declaration of 1975, as revised in 2008, typical ethics provisions of the Ministry of Public Health of Ukraine 66 dated February 13, 2006. The study protocol was approved by the Ethics Committee on Biomedical Research of BSMU (protocol biological medical ethical review No. 7 dated May 17, 2018, chairmen – MD, professor Zamorskyi I.I).

### Consent to participate

Written informed consent was obtained from the patients.

### Personal thanks

The authors thank all participants of this study.

### Authorship

LS, MS contributed to the study conception and design, data extraction, and the drafting of the manuscript; MS, AS equally contributed to data extraction, quality assessments, analysis of data, and the drafting of the manuscript. VD, OK, RS, IS contributed to data extraction, quality assessments, and data analysis. MS, VS, VD, RS, AS contributed to quality assessments and analysis of data. LS, NM, OB, IS contributed to the analysis and interpretation of the descriptive data. M.S. (corresponding author) contributed to the study conception and design and the critical revision. All authors have read and approved the final manuscript.
